# Metastatic squamous cell carcinoma of colon from esophageal cancer

**DOI:** 10.1186/s40164-017-0069-2

**Published:** 2017-04-18

**Authors:** Natasha Garg, Constance Stoehr, Yan Shi Zhao, Heather Rojas, Chung-Tsen Hsueh

**Affiliations:** 1 Department of Hematology-Oncology, North Shore University Hospital and Long Island Jewish Medical Center, Northwell Health System, Manhasset, NY 11030 USA; 2 AIS Cancer Center, 2620 Chester Ave, Bakersfield, CA 93301 USA; 3 Division of Gastroenterology and Hepatology, Department of Internal Medicine, Loma Linda University, Loma Linda, CA 92354 USA; 4 Department of Pathology and Human Anatomy, Loma Linda University, Loma Linda, CA 92354 USA; 5 Division of Medical Oncology and Hematology, Department of Internal Medicine, Loma Linda University, 11175 Campus Street, CSP 11015, Loma Linda, CA 92354 USA

**Keywords:** Esophageal squamous cell carcinoma, Colonic metastasis, Radiation

## Abstract

**Background:**

Esophageal cancer including squamous cell carcinoma (SCC) and adenocarcinoma represents 4% of all cancers in the United States. Patients with esophageal cancer frequently present with locally advanced disease, and about 40% of patients have evidence of metastatic disease on presentation. Common sites of metastasis include liver, lung and bone. Here, we present a rare case of colonic metastasis from primary esophageal SCC.

**Case presentation:**

A 60-year-old Caucasian male with a history of 20-pack-year cigarette smoking received surgery and adjuvant chemoradiotherapy for locally advanced SCC of larynx. Approximately 9 months later, he developed dysphagia, and found to have a esophageal SCC in the mid-esophagus with regional lymph node involvement. He underwent chemoradiation treatment with good response and improved symptoms but declined subsequent surgical resection for esophageal cancer. About 1 year after the diagnosis of esophageal cancer, he developed blood streaked bowel movement and severe anemia. Colonoscopy showed a 3-cm mass in the proximal ascending colon; biopsy showed metastatic SCC, consistent with metastasis from esophageal primary. He subsequently received palliative radiation to the ascending colon metastatic tumor with improvement of anemia, and remained transfusion independent for more than 3 months.

**Conclusions:**

Colonic metastasis from esophageal SCC is rare, and associated with poor prognosis. There are no definite features in terms of location, histological differentiation etc. that contribute to colonic metastasis from primary esophageal SCC. The goal of treatment is palliative and data from our and other case reports support the use of chemotherapy and radiation for symptom improvement and disease control.

## Background

Esophageal cancer including squamous cell carcinoma (SCC) and adenocarcinoma represents 4% of all cancers in the United States; in 2016, it is estimated approximately 16,000 new cases and more than 15,000 deaths as a result of this aggressive disease [1]. Globally, esophageal cancer affects more than 450,000 peoples, and is the eighth most common cancer [2]. The incidence of esophageal cancer is rising rapidly with adenocarcinoma being more than SCC in the Western world; obesity, gastroesophageal reflux disease and Barrett’s esophagus have shown to increase the risk of esophageal adenocarcinoma [3]. Patients with esophageal cancer frequently present with locally advanced disease, and about 40% of patients have evidence of metastatic disease on presentation. Common sites of metastasis include liver, lung and bone [4]. Here, we present a rare case of colonic metastasis from primary esophageal SCC.

## Case presentation

In July 2009, a 60-year-old Caucasian male with hypertension and dyslipidemia presented with neck stiffness and right neck mass for 3 months. The patient had a 20-pack year history of smoking and moderate alcohol intake. Initial ENT evaluation with direct laryngoscopy showed some fullness in the right base of the tongue without any abnormality at the larynx or pyriform fossa. CT scan of neck demonstrated a 3.8-cm necrotic mass in the right neck. Ultrasound guided biopsy of the right neck mass was consistent with malignant cells favoring poorly differentiated SCC. Patient was referred to our center for further work up. Repeat direct laryngoscopy showed a suspicious lesion at the right aryepiglottic fold. He underwent CT scan of chest, abdomen and pelvis plus PET-CT, which showed a right neck mass (4.2 cm × 3.5 cm) medial to the sternocleidomastoid muscle. A diagnosis of metastatic SCC of the right cervical lymph node with likely primary lesion at the base of the tongue or larynx was made.

Patient underwent right supra-glottis CO_2_ laser laryngectomy and right modified radical neck dissection in September 2009. Pathology from the right supra-glottis laryngectomy showed 0.6 cm tumor, which was consistent with moderately to poorly differentiated SCC with clear margins. Right neck dissection showed 3 out of 21 lymph nodes positive for metastatic SCC with the largest being 3 cm in greatest dimension. There was an extra-nodal extension of the tumor. The final diagnosis was T1N2bM0 (Stage IVA) right supra-glottis laryngeal SCC. Patient received adjuvant treatment with chemoradiotherapy completed in January 2010. Two months after completion of adjuvant treatment, the fiber-optic laryngoscopy and CT scan of neck showed no evidence of disease.

In October 2010, patient developed dysphagia, and examination of esophagogastroduodenoscopy and showed a stricture at 31 cm, a friable mass at 31–34 cm, Barrett’s mucosa noted from 34 to 39 cm, and the diaphragm at 39 cm from the incisors, respectively. Endoscopic ultrasound was done but the scope could not be passed through the stricture, but showed a mass at about 24 cm from the incisors in the paraesophageal area just below the aortic arch. Fine needle aspiration biopsy of the esophageal stricture and mediastinal mass showed poorly differentiated SCC, positive for p63 and CK 5/6 by immunohistochemical study. Imaging study with PET-CT and CT scan of neck, chest, abdomen and pelvis confirmed locally advanced esophageal cancer with mediastinal lymph node involvement. He underwent chemoradiotherapy with weekly paclitaxel and carboplatin, completed in February 2011. A follow-up CT scan of chest in June 2011 indicated a good treatment response with tumor shrinkage. He declined surgery after completion of chemoradiotherapy.

**Fig. 1:**
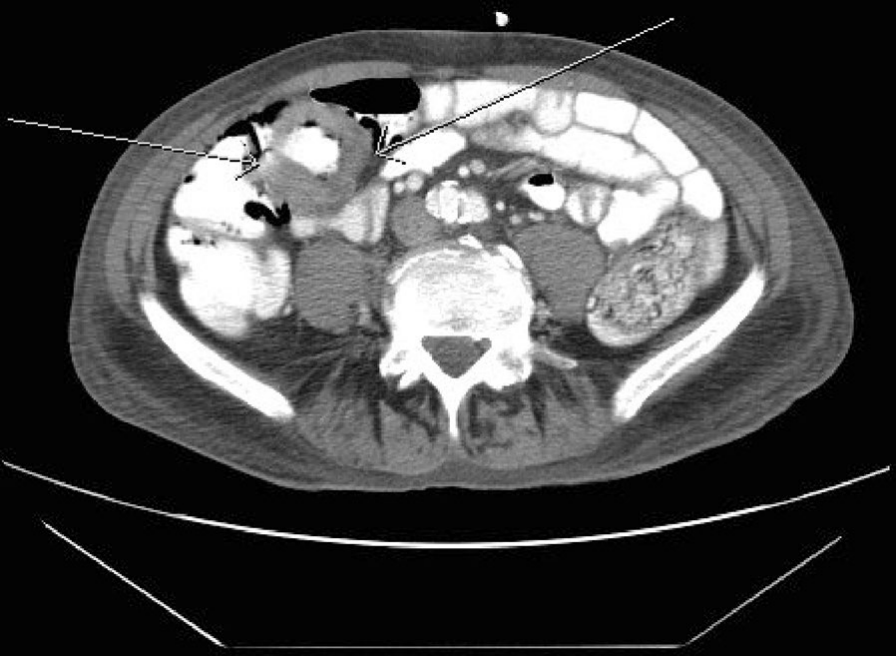
A mass at the ascending colon demonstrated on CT scan of abdomen and pelvis. The *arrow* showed a circumferential thickening of a segment of ascending colon due to colonic mass

**Fig. 2:**
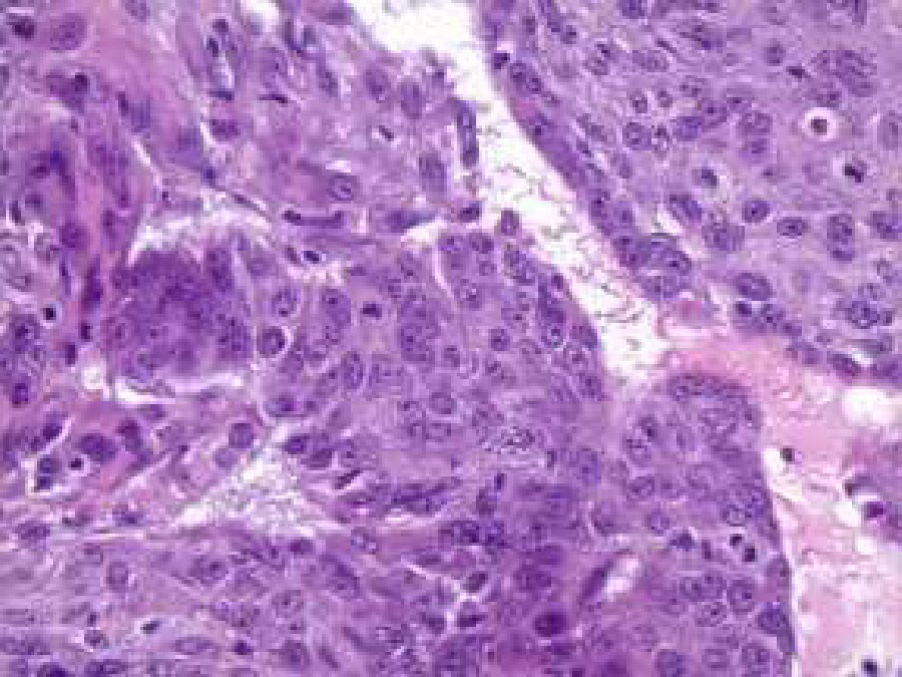
Microscopic finding of the colon tumor. Biopsy by colonoscopy showed poorly differentiated squamous cell carcinoma with dense cytoplasm, irregular nuclear contours, moderate pleomorphism and prominent nucleoli (hematoxylin and eosin stain, ×40). Additional immunohistochemical study and comparison to prior tumor histology indicated metastatic rather than a primary colorectal carcinoma

In November 2011, he developed blood streaked bowel movement and severe anemia with hemoglobin of 5.9 g/dL. He underwent CT scan of abdomen and pelvis and colonoscopy, and was found to have a 3-cm mass in the proximal ascending colon (Fig. 1). The biopsy showed metastatic SCC (Fig. 2). Immunohistochemical study showed the tumor was strongly positive for CK 5/6, with foci of weak staining for p63 and negative staining for CK20 or CDX2, which was most consistent with a metastatic lesion rather than a primary colorectal carcinoma. He subsequently received palliative radiation (3000 cGy) to the ascending colon cancer with improvement of anemia, and remained transfusion independent for more than 3 months. In January 2012, he also received palliative radiation for brain metastasis. He was offered palliative chemotherapy but he refused. He entered hospice in May 2012, and passed away shortly afterwards.

## Discussion

Our patient initially underwent definitive surgery and adjuvant chemoradiotherapy for locally advanced laryngeal SCC; the follow-up surveillance examination including endoscopic and CT scan examination showed no evidence of disease. However approximately 10 months after adjuvant treatment, he developed a second primary tumor (SPT), esophageal SCC. It has been shown that patients surviving laryngeal cancer treatment have increased risk of SPT, usually in the aerodigestive tract; the cumulative risk of developing a SPT is found to be 26% at 10 years and 47% at 20 years [5]. Field cancerization, also known as condemned mucosal syndrome, has been well characterized in patients with SCC of head and neck (SCCHN) [6, 7]. This concept of field effect in cancer postulates the accumulation of multiple molecular alterations from long-term carcinogen exposure in the upper aerodigestive tract leads to an increased risk of malignant transformation in the whole tissue region. Active tobacco use has been shown to increase the risk of SPT in patients with SCCHN [8]. In a phase III study, chemoprevention with isotretinoin reduced the incidence of SPT in patients with SCCHN, but the decrease in SPT did not lead to an increase in overall survival [9, 10]. Therefore, life style modification including smoking cessation, close surveillance and imaging study based on symptoms and clinical examination but not chemoprevention are recommended in SCCHN patients with increased risk of SPT [11].

Esophageal cancer is among the top 10 leading cause of cancer-related mortality and common cancer worldwide. There is a decreasing incidence of esophageal SCC but increasing incidence of esophageal adenocarcinoma in the Western world. Most patients present with locally advanced disease which requires combined-modality treatment including chemoradiotherapy with or without surgery. Neoadjuvant chemoradiation followed by surgery has been shown to improve local control, disease-free survival and overall survival compared to surgery alone [12]. For patients with locally advanced disease and unfit for surgery, the addition of concurrent chemotherapy to radiotherapy provides a chance of long-term survival [13]. The liver, lung and the bones are common sites of metastases in esophageal cancer.

Our patient declined surgery after chemoradiotherapy for locally advanced esophageal SCC, and developed metastatic disease to ascending colon with severe hematochezia and anemia about 9 months afterwards. His symptoms improved after palliative radiation to colonic metastasis, and remained transfusion independent for more than 3 months. He also developed brain metastasis and received brain radiotherapy before succumbing to esophageal cancer about 6 months after being diagnosed with metastatic disease.

The predominant histology type of primary colorectal cancer is adenocarcinoma; less common ones include neuroendocrine tumor, lymphoma, sarcoma and SCC [14]. The incidence of primary colorectal SCC is about 0.4% of colorectal cancers, and more than 90% of cases are at the rectum which may be associated with human papillomavirus infection and shares similar carcinogenesis process as anal canal SCC [15]. Metastatic involvement of the colon is a rare occurrence and is extremely rare from primary extra-abdominal tumors. There are case reports of metastatic colon cancer from primary malignancy such as melanoma, SCC of the lung, gastric adenocarcinoma, and invasive ductal breast carcinoma [16–18]. To this date, there are three case reports from Asia published in English literature describing colonic metastasis from primary esophageal SCC [19–21]. All reported cases were identified as synchronous metastasis to colon from primary esophageal SCC (Table 1). Our patient is the first case in Western world with metachronous colonic metastasis from primary esophageal SCC. The colonic metastasis was unlikely from laryngeal cancer due to the timing and characteristic of metastasis, but most likely due to retrograde lymphatic spreading from esophageal SCC [22].

**Table 1:** Published cases of colonic metastasis from esophageal squamous cell carcinoma

Authors and published year	Location	Timing	Symptom	Treatment	Survival from colonic metastasis
Karwasra et al. 2002 [19]	Transverse	Synchronous	None	Resection	Unknown
Iwase et al. 2004 [20]	Sigmoid	Synchronous	Bleeding	Chemotherapy	1 year
Shimada et al. 2014 [21]	Transverse	Synchronous	None	Resection	2.5 months
Garg et al. 2017 (the present report)	Ascending	Metachronous	Bleeding	Radiation	6 months

For patients with metastatic esophageal SCC, the goal of treatment is palliative to improve quality of life and survival, which requires multi-disciplinary approach including pain and symptom management. Our patient declined chemotherapy but received colonic radiation with improved symptoms and independency of blood transfusion lasting for 3 months. Palliative chemotherapy has yet to demonstrate survival improvement compared to best supportive care, but is frequently used with platinum-based regimen [23]. Targeted therapy such as inhibitors of angiogenesis and epidermal growth factor receptor provides no additional benefit to chemotherapy [24, 25]. Immunotherapy with PD-1 inhibitor has demonstrated promising activity in early-phase study, and may eventually change the treatment landscape for metastatic esophageal SCC [26].

## Conclusions

Colonic metastasis from esophageal SCC is rare, and associated with poor prognosis. There are no definite features in terms of location, histological differentiation etc. that contribute to colonic metastasis from primary esophageal SCC. The goal of treatment is palliative and data from our and other case reports support the use of chemotherapy and radiation for symptom improvement and disease control.

## Abbreviations

SCC: squamous cell carcinoma
SPT: second primary tumor
SCCHN: squamous cell carcinoma of head and neck
